# The predictive value of proximal femoral CT attenuation in intramedullary fixation failure of intertrochanteric fractures: a retrospective cohort analysis

**DOI:** 10.1007/s11657-025-01651-z

**Published:** 2025-12-19

**Authors:** Kaifeng Ye, YouLiang Hao, Yanlei Dong, Yuanyu Hu, Junbo Qi, Yutian Luo, Yun Tian

**Affiliations:** 1 Department of Orthopedics, Peking University Third Hospital, No. 49 North Garden Road, Haidian District, Beijing, 100191 China; 2 Engineering Research Center of Bone and Joint Precision Medicine, No. 49 North Garden Road, Haidian District, Beijing, 100191 China

**Keywords:** Intertrochanteric fracture, CT value, Implant failure, Risk factors

## Abstract

***Summary*:**

This study investigated whether low hip bone density (as measured by CT scans) can predict implant failure in hip fracture patients undergoing nail fixation. Based on an analysis of 143 cases, patients with CT Hounsfield units below 27.6 HU had a significantly higher risk of failure. This preoperative metric offers a quantifiable tool to guide surgical decisions and improve outcomes in patients with fragile bones.

**Purpose:**

To determine whether reduced proximal femoral computed tomography (CT) Hounsfield units constitute an independent risk factor for internal fixation failure after intramedullary nail fixation for intertrochanteric fractures.

**Methods:**

In this retrospective cohort study, patients who underwent intramedullary fixation for intertrochanteric fractures were categorized into fixation failure and non-failure groups on the basis of follow-up outcomes. Demographic, clinical, and imaging parameters were analyzed, with a focus on proximal femoral CT values measured in Hounsfield units (HU). Logistic regression models were employed to identify independent risk factors, while receiver operating characteristic (ROC) curve analysis established optimal CT value thresholds for predicting failure.

**Results:**

Among the 143 enrolled patients, 21 (14.7%) developed fixation failure. Compared with the control group, the failure group exhibited significantly lower proximal femoral CT Hounsfield units (14.6(19.4) HU vs. 46.9(37.3) HU; *P* < 0.001). Multivariate analysis demonstrated that each 1 HU increase in CT value reduced the risk of failure by 5.1% (*P* < 0.001). ROC analysis revealed 27.6 HU as the optimal predictive threshold, yielding 81% sensitivity and 86% specificity.

**Conclusion:**

Proximal femoral CT Hounsfield units below 27.6 HU significantly predict internal fixation failure in patients with intertrochanteric fractures treated with intramedullary nails, providing a quantifiable preoperative risk assessment tool.

## Introduction

Intertrochanteric fractures account for approximately 40% of hip fractures and represent a major cause of mortality and disability in the elderly population [1], significantly compromising both life expectancy and quality of life [2, 3]. Intramedullary nailing has become the standard surgical treatment for these fractures; however, postoperative implant failure remains a devastating complication, exposing patients to additional surgical burdens while substantially increasing risks of functional decline and mortality [4].

Osteoporosis, characterized by reduced bone mineral density (BMD) and deteriorated trabecular architecture, plays a dual role in intertrochanteric fractures. It not only predisposes individuals to low-energy fractures [5, 6] but also increases their susceptibility to fixation failure through compromised implant anchorage [7, 8]. Preoperative bone quality assessment is important for optimizing surgical planning and mitigating complications. However, clinical implementation faces substantial challenges: approximately 80% of osteoporotic patients remain undiagnosed prior to their first fragility fracture [8, 9], due to the limited accessibility of dual-energy X-ray absorptiometry (DXA), the WHO-endorsed gold standard for osteoporosis diagnosis, coupled with DXA confounders and patient reluctance regarding radiation exposure [8–12]. The preoperative evaluation of bone quality could, to a certain extent, help the surgeons to make better surgical strategies and postoperative rehabilitation plans.

Emerging evidence suggests that proximal femoral CT values from routine preoperative CT scans strongly correlate with DXA-derived BMD [13, 14]. Given that hip CT is mandatory for preoperative planning in patients with intertrochanteric fractures, proximal femoral CT Hounsfield units provide a pragmatic opportunity for opportunistic osteoporosis screening without an additional imaging burden. Nevertheless, the direct association between proximal femoral CT value and the risk of intertrochanteric fracture implant failure remains undefined. Current literature predominantly focuses on biomechanical correlations and lacks clinical validation in real-world surgical cohorts. This study aims to bridge this critical knowledge gap by investigating whether reduced proximal femoral CT Hounsfield units serve as an independent predictor of intramedullary fixation failure in patients with intertrochanteric fractures. Our findings may establish a novel preoperative decision-making framework for implant selection and patient-specific risk mitigation.

## Materials and methods

### Study design

This retrospective cohort study included patients who underwent surgical treatment for intertrochanteric fractures at our institution between January 2010 and December 2017. The inclusion and exclusion criteria are detailed in Table 1. Participants were stratified into two groups on the basis of postoperative outcomes: case group, patients with radiographic evidence of internal fixation failure during follow-up; and control group, patients who achieved uneventful fracture union without complications at a minimum of 1-year follow-up.

**Table 1:** Inclusion and exclusion criteria

Inclusion	Exclusion
• AO 31-A2 type intertrochanteric fracture	• Patients with open fractures
• Fresh, closed fracture that underwent closed reduction and internal fixation	• Patients with multiple injuries
• Internal fixation performed using intramedullary nail	• Patients with pathological fractures
• Regular postoperative imaging follow-up until fracture healing or internal fixation failure occurred	• Patients with subtrochanteric fractures
• For patients without internal fixation failure, follow-up duration exceeds 1 year	• Patients with ASA scores greater than 3
• Preoperative or imaging performed within 6 months prior to injury that included CT scans of the proximal femur	• Patients with pre-existing functional impairments prior to injury
	• Patients with simultaneous bilateral intertrochanteric fractures
	• Patients who sustained contralateral lower limb fractures during follow-up
	• Patients with incomplete follow-up data
	• Patients without preoperative CT imaging of the proximal femur or those with imaging performed more than 6 months prior

### Data collection

Demographic, surgical, and imaging parameters were systematically extracted, including preoperative proximal femoral CT Hounsfield units (HU), fixation failure status (binary endpoint), and other characteristics such as age, sex, ASA classification, fracture laterality, injury-to-surgery interval, operative duration, intraoperative blood loss, hospitalization length, and integrity of posteromedial wall support.

### Protocol for proximal femoral CT value measurement

**Fig. 1:**
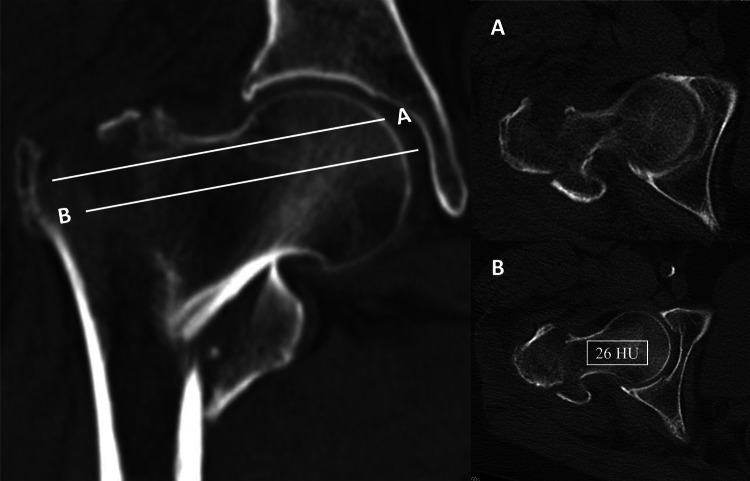
Methodology for measuring proximal femoral CT Hounsfield units: (A) reference axial slice (first slice; 0 mm); (B) targeted axial slice (second slice; 5 mm). This patient’s proximal femoral CT Hounsfield unit was 26 HU, and she eventually experienced varus deformity and delayed nonunion

All CT scans were acquired using standardized parameters (tube voltage, 120 kV; slice thickness, 5 mm). The CT scans that included the proximal femur consisted of abdominal CT, urinary system CT, pelvic CT, hip joint CT, and thigh CT scans. Proximal femur HU measurements were performed on PACS workstations following a validated methodology. Through preliminary research analysis, we found that the CT Hounsfield units of the second axial slice (5 mm) were most strongly correlated with those of the DXA [14]. Therefore, in this study, we measured only the CT Hounsfield units of the second axial slice. The first axial slice was defined as the section containing the femoral neck connected to the greater trochanter without the superior femoral neck cortex, while the second axial slice referred to the next one (5-mm interval between slices). Our measurement method was consistent with that of previous studies, specifically measuring the average CT Hounsfield units of the area of interest (ROI) with the maximum range of trabecular bone in the axial view of the proximal femur, which includes the femoral neck and intertrochanteric region [14] (Fig. 1).

### Outcome evaluation

The primary outcome of this study was whether internal fixation failure occurred after surgery for intertrochanteric fractures. This determination was primarily based on the imaging examinations conducted during the follow-up of patients. Patients who exhibited any of the following conditions were classified as having experienced internal fixation failure: (1) varus deformity (during follow-up); (2) pull-out of the lag screw; (3) breakage of the internal fixation device; (4) occurrence of the Z-effect phenomenon, i.e., screw loosening; (5) periprosthetic fracture; and (6) failure of fracture healing [15].

### Surgical technique

All patients underwent a specialized discussion before surgery, which involved at least four experienced trauma orthopedic experts. The surgeries were performed by skilled trauma orthopedic surgeons, with anesthesia administered using either spinal anesthesia or general anesthesia. The procedures were carried out on an orthopedic traction table, with continuous assistance from bedside X-ray fluoroscopy throughout the operation. Once X-ray fluoroscopy confirmed that the closed reduction position was satisfactory, the intramedullary fixation device for intertrochanteric fractures was inserted under fluoroscopic guidance. The surgical process for all patients was smooth, and no intraoperative complications occurred.

### Statistical analysis

Data management and analysis were performed using Excel 2010 (Microsoft) and SPSS 24.0 (IBM), respectively. For continuous variables, we employed the Shapiro-Wilk test to assess the normality of the data. If the data were normally distributed, we used *t* tests for comparative analysis; if not, we used nonparametric rank-sum tests (Mann-Whitney *U* tests) for comparison. For categorical data, we used chi-square tests for comparative analysis. For parameters identified as potential risk factors for internal fixation failure during the analysis, we conducted logistic regression analysis, including the number of proximal femur CT Hounsfield units and all potential risk factors, to verify whether the number of proximal femur CT Hounsfield units was an independent risk factor for internal fixation failure. In all analyses, we considered a *p*-value of < 0.05 to indicate statistical significance.

## Results

### Baseline data

**Fig. 2:**
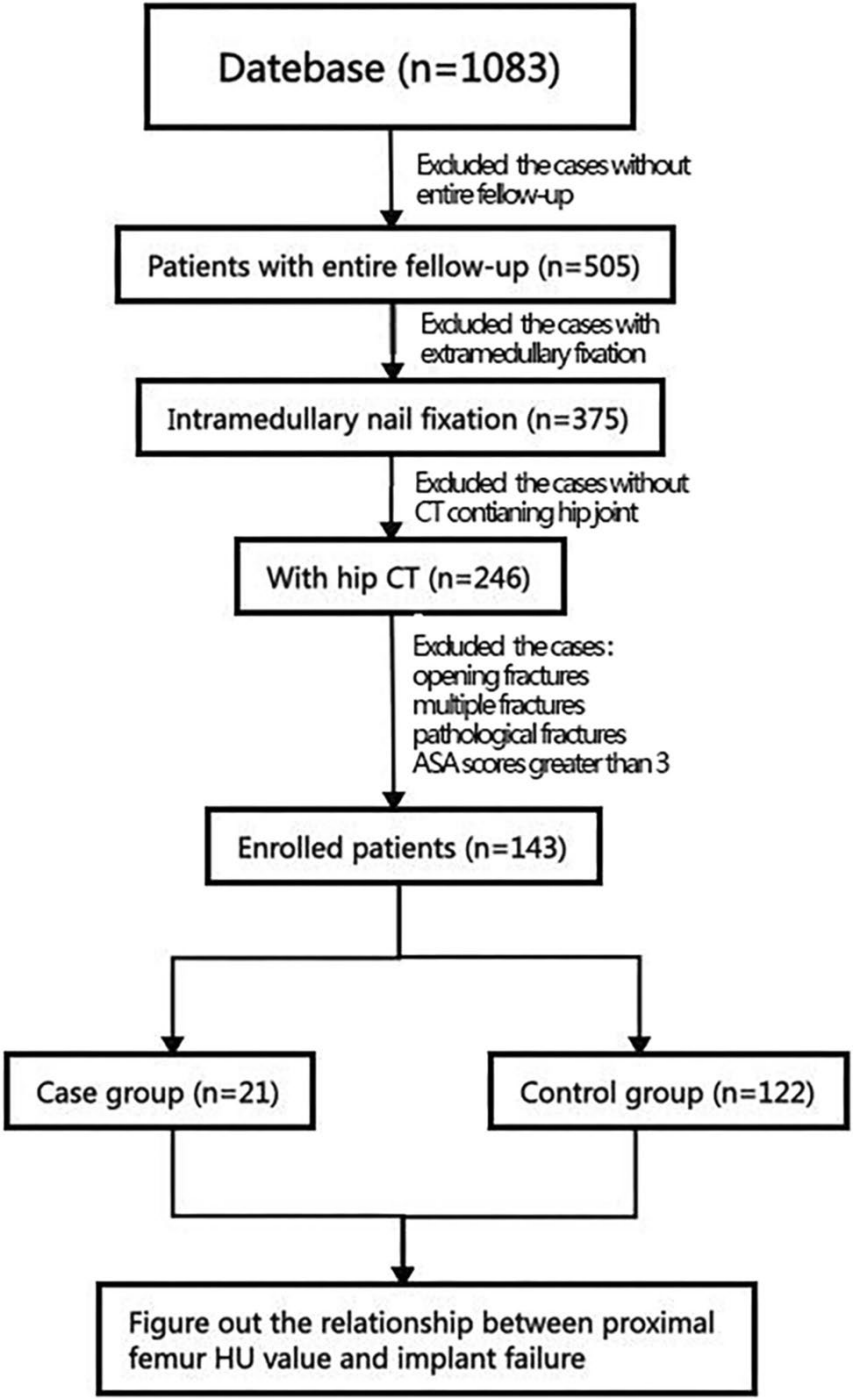
Flowchart displaying subject selection based on inclusion and exclusion criteria

From January 2010 to December 2017, 1083 patients underwent intramedullary fixation surgery at our hospital, and 66 patients experienced failure during follow-up; the actual failure rate was 6.1%. After stringent inclusion and exclusion criteria were applied, 143 patients (53 males, 90 females) were enrolled in the final cohort (Table 1, Fig. 2). The cohort had a mean of 76.5 ± 11.8 years and a mean number of proximal femoral CT Hounsfield units of 42.8 ± 27.3 HU. Fracture laterality was balanced (left, 52.4%; right, 47.6%), with compromised posteromedial wall integrity observed in 56.6% of the patients, and the distribution of ASA classifications was Grade I (13.3%, *n* = 19), II (56.6%, *n* = 81), and III (30.1%, *n* = 43) (Table 2).

**Table 2:** Comparison of fixation failure or non-failure patients

Variables	Overall	Case group (*n* = 21)	Control group (*n* = 122)	*X*^2^/*Z*-value	*P*-value
Categorical data					
Sex				1.854	0.173
Male	53	5 (23.8%)	48 (39.3%)		
Female	90	16 (76.2%)	74 (60.7%)		
Fracture side				2.033	0.154
Left	75	8 (38.1%)	67 (54.9%)		
Right	68	13 (61.9%)	55 (45.1%)		
ASA (I: II: III)				4.221	0.121
I	19	1 (4.8%)	18 (14.8%)		
II	81	10 (47.6%)	71 (58.2%)		
III	43	10 (47.6%)	33 (27.0%)		
Medial wall support				5.923	**0.015**
Have	62	4 (19.0%)	58 (47.5%)		
Not have	81	17 (81.0%)	64 (52.5%)		
Continuous data					
Age (years)	76.51 ± 11.75	81.0 (10.0)	79.0 (11.0)	−1.264	0.206
Gender (male: female)	53:90	5:16	48:74		0.173
Injury-to-surgery interval (days)	4.0 (4.0)	3.0 (7.0)	4.0 (4.0)	−0.349	0.727
Operative duration (min)	70.0 (40.0)	74.0 (108.0)	70.0 (37.0)	−1.109	0.267
Intraoperative blood loss	100.0 (100.0)	150.0 (295.0)	90.0 (100.0)	−2.426	**0.015**
Hospitalization length	6.0 (4.0)	6.0 (6.0)	6.0 (4.0)	−1.168	0.243
Proximal femur CT value (HU)	42.8 ± 27.3	12.6 (19.4)	46.9 (37.3)	−5.256	** < 0.001**

Statistically significant values are highlighted in boldface

### Univariate analysis of the two groups

During a median follow-up of 26 months (range 12–48 months), 21 patients (14.7%) experienced fixation failure at a median time of 5 months after surgery. Failure modes included varus collapse (52.4%, *n* = 11), screw cut-out (23.8%, *n* = 5), peri-implant fracture (9.5%, *n* = 2), nonunion (9.5%, *n* = 2), and implant breakage (4.8%, *n* = 1). As shown in Table 2, compared with the controls, the case group (failure group) had significantly lower proximal femoral CT Hounsfield units (14.6 (19.4) HU vs. 46.9 (37.3) HU; *P* < 0.001), a greater prevalence of posteromedial wall defects (81.0% vs. 52.5%; *P* = 0.015), and greater intraoperative blood loss (150.0 (295.0) mL vs. 90.0 (100.0) mL; *P* = 0.015). No significant differences were observed in terms of age, sex distribution, ASA classification, or fracture laterality (*P* > 0.05).

### Logistic regression analysis of risk factors

In the univariate analysis comparing the case and control groups, statistically significant differences were identified in three parameters: the integrity of the posteromedial wall, intraoperative blood loss, and proximal femoral CT Hounsfield units. To further investigate whether low proximal femoral CT Hounsfield units constitute an independent risk factor for fixation failure in patients with intertrochanteric fractures, these three significant variables, along with potential confounders, including age, sex, and fracture laterality, were incorporated into a logistic regression model. As shown in Table 3, after adjusting for other potential risk factors, the number of proximal femoral CT Hounsfield units remained significantly associated with fixation failure. Reduced CT Hounsfield units were identified as an independent risk factor for intramedullary fixation failure, with each 1 HU increase in CT Hounsfield units associated with a 5.1% reduction in failure risk (adjusted OR = 0.949; 95% CI, 0.920–0.979; *P* = 0.001).

**Table 3:** Logistic regression analysis of risk factors for fixation failure of intertrochanteric fractures

Variables	*P*-value	OR	95% CI
Sex	0.352	1.867	0.501–6.954
Age	0.477	1.030	0.950–1.117
Fracture side	0.611	1.356	0.420–4.381
Medial wall support	0.261	0.458	0.117–1.790
Intraoperative blood loss	**0.009**	1.004	1.001–1.007
Proximal femur CT value (HU)	**0.001**	0.949	0.920–0.979

Statistically significant values are highlighted in boldface

### ROC curve analysis

**Fig. 3:**
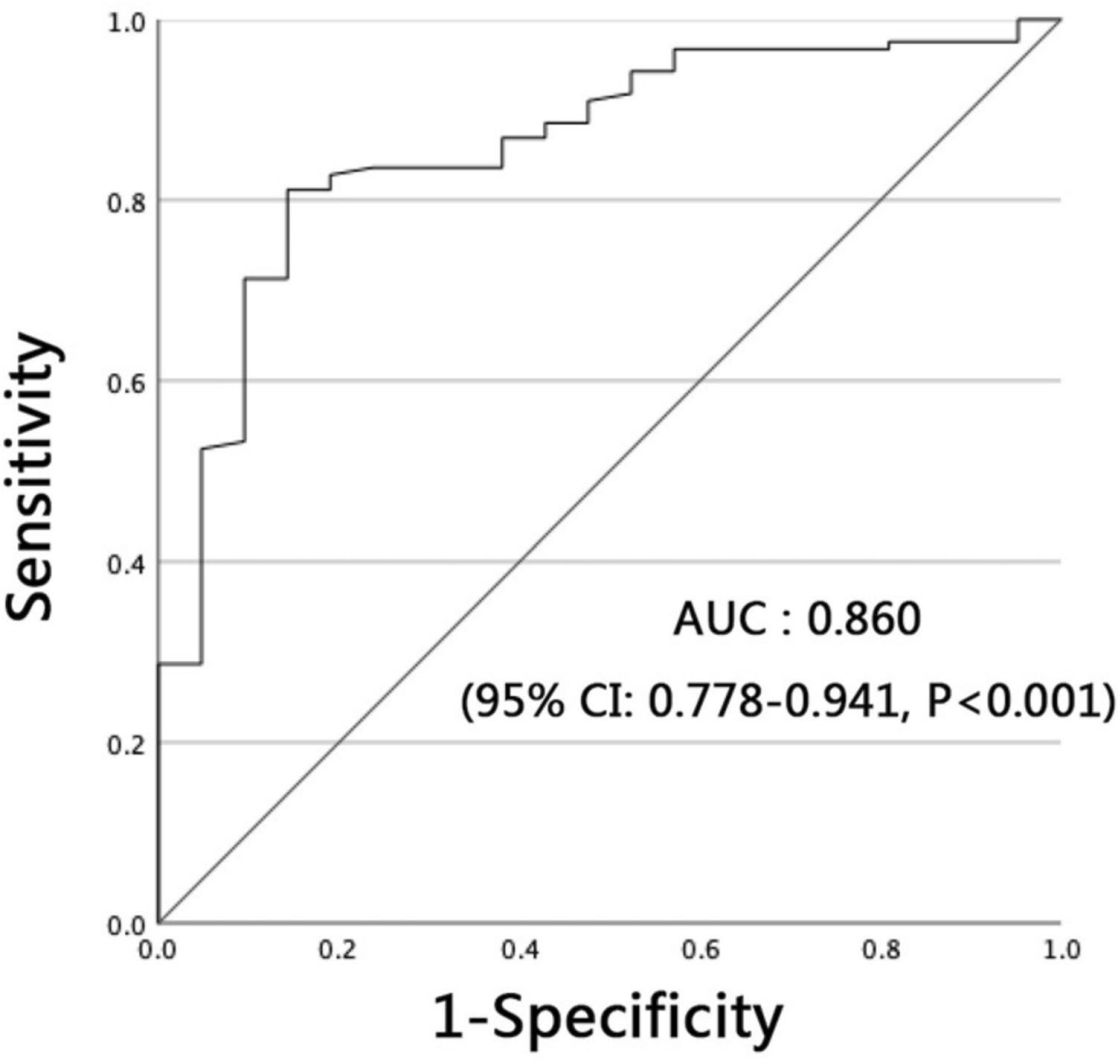
ROC curve analysis of the risk of internal fixation failure in patients with intertrochanteric fractures using proximal femoral CT Hounsfield units

The aforementioned analysis demonstrated that lower proximal femoral CT Hounsfield units are associated with a higher risk of internal fixation failure after intramedullary fixation for intertrochanteric fractures. To investigate the critical threshold of proximal femoral CT Hounsfield units that warrants clinical concern, we conducted ROC curve analysis to evaluate the efficacy of using proximal femoral CT Hounsfield units in assessing the risk of internal fixation failure. The ROC curve plotted in Fig. 3 yielded an area under the curve (AUC) of 0.860 (95% CI, 0.778–0.941; *P* < 0.001). The maximum Youden index for predicting internal fixation failure risk using proximal femoral CT Hounsfield units was 0.67, corresponding to an optimal cutoff value of 27.6 HU. At this threshold, the sensitivity and specificity for identifying internal fixation failure were 81% and 86%, respectively. Furthermore, the cutoff value with high sensitivity of 90% (high specificity of 90%) to identify internal fixation failure was 13.8 HU (36.4 HU).

## Discussion

The number of patients with intertrochanteric fractures, which are common sites of pathological fractures in osteoporotic patients, is steadily increasing annually, and these fractures have emerged as a global health concern that threatens the quality of life and life expectancy of elderly people. For patients requiring internal fixation for intertrochanteric fractures, the severity of osteoporosis is a critical factor influencing the risk of postoperative internal fixation failure. However, due to the limited accessibility and confounders of dual-energy X-ray absorptiometry (DXA), the current gold standard for osteoporosis diagnosis, a significant number of osteoporotic patients remain undiagnosed and untreated, posing substantial challenges in clinical management [3, 16–21]. Recently, our research validated the correlation between proximal femoral CT Hounsfield units and DXA results for osteoporosis diagnosis [14]. This enables surgeons to assess bone quality preoperatively with routine hip CT scans.

In this study, the mean proximal femoral CT Hounsfield units of both the case and control groups were significantly lower than the 67 HU threshold proposed by our recent study for screening osteoporotic patients using proximal femoral CT Hounsfield units [14]. These findings further validate the reliability of proximal femoral CT Hounsfield units in assessing bone quality. Previous studies have also identified severe osteoporosis as a risk factor for internal fixation failure in patients with intertrochanteric fractures [6, 22–24]. However, as noted earlier, many osteoporotic patients remain undiagnosed, and most do not undergo bone density evaluations prior to their first fracture [8, 9, 17]. After a fracture, emergency constraints often preclude preoperative bone density testing. Subsequent research exploring quantitative computed tomography (QCT) has confirmed that low QCT-derived bone density is correlated with postoperative fixation failure in hip fracture patients [25–27]. Nevertheless, QCT requires specialized phantoms and complex software, limiting its widespread adoption [28–30]. In contrast, the hip CT scans used in our study were routinely performed for preoperative assessment. Therefore, our findings suggest that clinicians can utilize proximal femoral CT measurements to preliminarily evaluate bone quality in patients with intertrochanteric fractures without recent bone density assessments and guide the selection of appropriate fixation strategies. For patients with CT Hounsfield units below 27.6 HU, more robust fixation methods (e.g., cement-augmented screws) [31–33] should be prioritized, with a heightened emphasis on postoperative antiosteoporosis therapy [34].

Varus deformity, a common complication after internal fixation for intertrochanteric fractures, may necessitate revision surgery in severe cases [35, 36]. Consistent with previous studies, our research identified varus deformity as the most frequent complication in cases of failure of internal fixation for intertrochanteric fractures. To date, no literature has explicitly addressed the relationship between osteoporosis and postoperative varus deformity in patients with intertrochanteric fractures. Insights from related studies on knee osteoarthritis suggest that osteoporotic patients—particularly those with low trabecular bone mineral density (BMD) in the medial femoral condyle and the medial tibial plateau—exhibit reduced resistance to stress during lower limb force transmission, thereby increasing their risk of varus deformity [37, 38]. Additionally, Liang et al. demonstrated through finite element analysis of intramedullary fixation for intertrochanteric fractures that the femoral calcar region and the locking interface between the intramedullary nail and the screws bear the highest stress loads [39]. These structures collectively resist varus forces during stress transmission. In intramedullary fixation systems, where screws and the main nail form an angular fixation configuration, the screw tip becomes a stress concentration point that counteracts varus forces due to lever mechanics. Therefore, the lower the number of proximal femur CT Hounsfield units, the greater the loading pressure on the screw tip, and the greater the risk of varus deformity. Prior studies have also confirmed that enhancing screw resistance to varus loads effectively reduces the risk of deformity [40, 41].

Another common complication observed in this study was screw cut-out, which frequently coexists with varus deformity. In recent years, increasing attention has been given to screw cut-out following the internal fixation of intertrochanteric fractures. Our findings align with those of previous studies, which demonstrated a heightened risk of screw cut-out in patients with severe osteoporosis [42, 43]. In the design of the Proximal Femoral Nail Antirotation (PFNA) system, the lag screw is intended to compact trabecular bone during insertion, thereby enhancing screw anchorage strength and improving fixation stability in osteoporotic bone [44]. However, we hypothesize that in patients with severe osteoporosis, diminished trabecular bone density around the screw may result in insufficient anchorage even under full compression, leading to fixation failure. Finite element analyses by Liang et al. further revealed the screw tip as a critical stress concentration zone, attributing this phenomenon to the mismatch in the elastic modulus between the screw and surrounding trabecular bone [39]. When localized stress exceeds the ultimate strength of osteoporotic trabecular bone, microfractures may propagate, ultimately leading to screw cut-out. While PFNA remains the preferred fixation method for osteoporotic intertrochanteric fractures due to its reduced failure risk [44], our study emphasizes that in patients with severe osteoporosis (preoperative proximal femoral CT Hounsfield units < 27.4 HU), supplemental stabilization strategies should be considered. These include optimizing screw trajectory and length or utilizing cement-augmented screws to mitigate fixation failure caused by compromised bone quality [31, 33, 39, 43].

Implant fracture is a relatively rare complication following intramedullary fixation of intertrochanteric fractures. Recent analyses of such cases propose a biomechanical mechanism analogous to lever mechanics: during force transmission between the screw and the main nail, the inferomedial locking interface acts as a fulcrum. When upper body load is applied to the screw tip, lever forces generated between the screw and the main nail may exceed the fatigue strength of the implant at this fulcrum, leading to fracture[45]. The literature further indicates that at the junction where the lag screw traverses the main nail, the cross-sectional area of the nail is reduced by approximately three-quarters, creating a critical stress concentration zone. This region serves as both the weakest point of the construct and the primary pathway for load transfer from the femoral neck to the shaft [46, 47]. Finite element analyses corroborate these findings, demonstrating a distinct “red zone” of stress concentration at the screw–nail interface during force transmission from the intramedullary nail to the femoral shaft. The stress magnitude in this region can exceed 1800 N, surpassing the fatigue limit of the implant and leading to catastrophic failure [48].

For patients undergoing intertrochanteric fracture surgery, achieving stable reduction at the fracture site is critical to ensure proper healing [49, 50]. Many studies have proposed that osteoporosis and instability at the fracture interface during weight-bearing—caused by various factors—are risk factors for nonunion and malunion [51–53]. Our findings align with these conclusions: in our finite element analysis, we observed that lower trabecular bone CT Hounsfield units in the femur were correlated with reduced overall model stability, thereby increasing the risk of nonunion or malunion. Periprosthetic fracture following internal fixation is a rare but severe postoperative complication, with catastrophic outcomes including mortality [54, 55]. Previous studies have highlighted the elevated risk of periprosthetic fractures in patients with severe osteoporosis [56], while others have demonstrated that enhanced postoperative antiosteoporosis therapy effectively mitigates this risk—a conclusion consistent with our findings [57]. The design of the Proximal Femoral Nail Antirotation (PFNA) system incorporates a 6-degree lateral bend at the transition from the greater trochanter to the femoral shaft, along with a variable nail diameter from the lesser trochanter level to the distal femoral isthmus. These features create uneven cortical contact within the medullary canal and a medial bias at the distal tip of the nail [58, 59]. Consequently, regions of concentrated cortical-nail contact experience disproportionately high stress. Prolonged loading beyond the fatigue limit of cortical bone in these areas may lead to periprosthetic fractures. Therefore, based on prior clinical evidence, patients with low preoperative CT Hounsfield units warrant intensified postoperative antiosteoporosis management to address these biomechanical vulnerabilities.

## Limitations

While this study provides valuable insights into the relationship between proximal femoral CT Hounsfield units and postoperative complications in patients with intertrochanteric fractures, several limitations should be acknowledged. First, the retrospective cohort design inherently carries risks of selection bias and unmeasured confounding variables, such as variations in surgical technique and, especially, a reduction in the number of enrolled patients. We need to standardize the process and conduct more rigorous research in the future. Second, the CT Hounsfield units’ threshold of 27.6 HU for predicting fixation failure, while statistically significant, requires external validation in multicenter prospective studies to confirm its universal applicability. Additionally, long-term follow-up data on patient outcomes, including functional recovery and late-onset complications, were not analyzed, leaving gaps in understanding the durability of interventions in osteoporotic populations. Finally, the control group was not obtained through pairing, and we need to establish a better trial in our future research. Addressing these limitations in future research could strengthen clinical translation and refine risk stratification strategies.

## Conclusions

Through univariate and logistic regression analyses of preoperative proximal femoral CT Hounsfield units in patients with intertrochanteric fractures, we identified low proximal femoral CT Hounsfield units as an independent risk factor for internal fixation failure. For every 1 HU increase in proximal femoral CT Hounsfield units, the risk of fixation failure decreased by a factor of 0.949 (OR = 0.949; 95% CI, 0.920–0.979; *P* = 0.001). Further ROC curve analysis demonstrated the utility of proximal femoral CT Hounsfield units in screening patients at high risk for postoperative fixation failure (AUC = 0.860; 95% CI, 0.778–0.941; *P* < 0.001). Patients with preoperative proximal femoral CT Hounsfield units below 27.6 HU (sensitivity 81%, specificity 86%) warrant heightened vigilance for fixation failure. For these cases, surgical strategies should prioritize reinforced fixation constructs—such as cement-augmented screws to enhance anchorage—in combination with intensified postoperative antiosteoporosis therapy and close clinical surveillance.

## Data Availability

The datasets generated during and/or analyzed during the current study are available from the corresponding author upon reasonable request.
